# Repeated Lake-Stream Divergence in Stickleback Life History within a Central European Lake Basin

**DOI:** 10.1371/journal.pone.0050620

**Published:** 2012-12-04

**Authors:** Dario Moser, Marius Roesti, Daniel Berner

**Affiliations:** Zoological Institute, University of Basel, Vesalgasse 1, Basel, Switzerland; Tuscia University, Italy

## Abstract

Life history divergence between populations inhabiting ecologically distinct habitats might be a potent source of reproductive isolation, but has received little attention in the context of speciation. We here test for life history divergence between threespine stickleback inhabiting Lake Constance (Central Europe) and multiple tributary streams. Otolith analysis shows that lake fish generally reproduce at two years of age, while their conspecifics in all streams have shifted to a primarily annual life cycle. This divergence is paralleled by a striking and consistent reduction in body size and fecundity in stream fish relative to lake fish. Stomach content analysis suggests that life history divergence might reflect a genetic or plastic response to pelagic *versus* benthic foraging modes in the lake and the streams. Microsatellite and mitochondrial markers further reveal that life history shifts in the different streams have occurred independently following the colonization by Lake Constance stickleback, and indicate the presence of strong barriers to gene flow across at least some of the lake-stream habitat transitions. Given that body size is known to strongly influence stickleback mating behavior, these barriers might well be related to life history divergence.

## Introduction

Speciation is often initiated by adaptation to ecologically distinct habitats in the face of gene flow [1]–[4]. This process is typically inferred from concurrent divergence in phenotypes and genetic marker frequencies across habitat transitions in the absence of physical dispersal barriers (e.g., [5]–[13]). Patterns aside, the actual mechanisms constraining gene flow in the early stages of ecological divergence generally remain poorly understood [4], [14], [15] (but see [16], [17]). At least partial reproductive isolation is often assumed to result directly from performance trade-offs associated with adaptive divergence. That is, divergence in ecologically important traits causes selection against maladapted migrants and hybrids between habitats [14], [18]–[20]. Further reductions in gene flow between populations can arise readily as indirect (correlated) consequences of adaptive divergence [4], [14], [21], [22], for instance when traits under ecological divergence also influence reproductive behavior [23]–[25]. Understanding speciation thus benefits greatly from a thorough understanding of adaptive divergence.

In animals, the traits receiving greatest attention in the context of ecological divergence and reproductive isolation are typically those related to resource acquisition and predator avoidance [14], [18]. By contrast, divergence in life history is less frequently considered as a driver of speciation, despite its potential to contribute to reproductive isolation at multiple levels simultaneously: first, adaptive divergence in life history traits in response to ecologically distinct habitats [26], [27] might directly reduce gene flow between populations through reduced performance of migrants and hybrids between the habitats. Second, life history divergence often involves shifts in reproductive timing, thereby potentially causing phenological assortative mating as a correlated response. Evidence of this mechanism exists but is mostly limited to insects (e.g., [28]–[30]; but see [31]). Third, life history divergence commonly involves body size shifts [26], [27]. Because body size is also frequently involved in sexual selection [32], life history divergence might drive sexual assortative mating as an additional correlated response. Finally, life history traits generally display higher levels of phenotypic plasticity than morphological, physiological, and behavioural traits, because the former represent greater targets for environmental perturbation [33], [34]. Life history shifts might thus follow rapidly upon the colonization of new habitats, and hence contribute to reproductive isolation well before genetically-based divergence in less plastic traits has occurred [35], [36].

The objective of this study is to initiate an investigation of life history divergence in a natural model system for studying speciation with gene flow – lake and stream populations of threespine stickleback fish (*Gasterosteus aculeatus* L.). Marine (ancestral) stickleback have colonized freshwater environments all across the Northern Hemisphere after the last glacial retreat, thereby establishing numerous evolutionarily independent population pairs residing in adjacent lake and stream habitats [37]–[46]. Lake and stream populations typically display predictable and at least partly genetically-based [39], [47], [48] divergence in morphological traits, presumably reflecting adaptation to distinct foraging environments. This phenotypic divergence often coincides with striking divergence in genetic markers on a small spatial scale [12], [46], [49], [50], indicating the presence of strong reproductive barriers associated with lake-stream transitions. The nature of these barriers, however, remains poorly understood (reviewed in [51]).

A contribution of life history divergence to reproductive isolation in lake-stream stickleback, through one or several of the mechanisms described above, is plausible because life history evolution is reported from other stickleback systems. This includes divergence in age at reproduction and reproductive investment within and among lake populations [52]–[56], and divergence in body size within and among lake populations [52], [53], [56]–[59] and between freshwater and marine stickleback [60], [61]. At least some of this divergence is partly genetically based [58], [62]. Furthermore, body size divergence is generally a strong contributor to mating isolation in the species ([59]–[61], [63]–[66]; but see [67]. Nevertheless, investigations of life history divergence in lake-stream stickleback are lacking.

Our study focuses on stickleback inhabiting contiguous lake and stream habitats within a single lake basin in Central Europe. We focus on multiple replicate lake-stream sample pairs to assess whether life history divergence has occurred repeatedly in a similar direction. Finally, we include nuclear and mitochondrial genetic marker data to search for signatures of habitat-associated barriers to gene flow, and to gain insight into the origin of lake and stream stickleback populations within the lake basin.

## Materials and Methods

### Stickleback Samples

**Figure 1:**
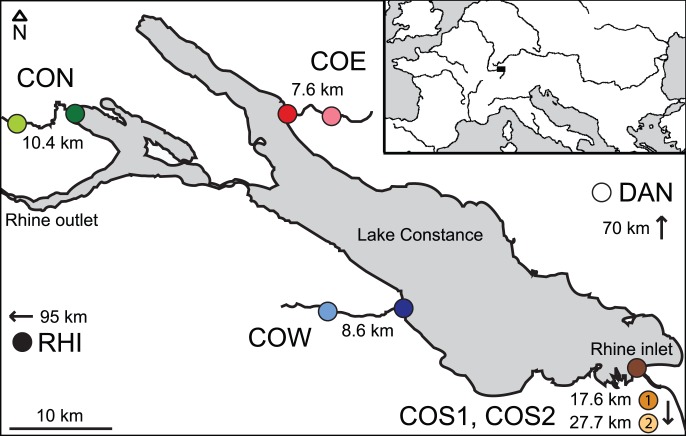
Geographical situation of the stickleback study sites. Shown are the five lake-stream stickleback pairs (‘systems’) in the Lake Constance basin (CON, COE, COS1, COS2, COW; colored circles, stream sites lighter), and the two solitary sample sites outside the basin (RHI, DAN; black and white circle). The black rectangle in the inset map locates the study area in Central Europe. Distances indicate the approximate water distance between the lake and stream site within each system, and the approximate map distance between Lake Constance and the solitary sample sites. Note that the COS1 and COS2 stream samples were not collected from the Rhine (the major inlet to Lake Constance), but from two small streams draining separately into Lake Constance. Further details on the samples and locations are given in Table 1.

The main focus of this life history investigation lies on stickleback in Lake Constance (LC) and its tributaries in Central Europe (Fig. 1, Table 1). The geographic distance between the different lake-stream pairs (‘systems’) was maximized to reduce the opportunity for gene flow among systems, and to provide phenotypic and genetic information representative of the entire lake basin. The systems include two lake-stream pairs subjected previously to an analysis of foraging morphology and population genetics (‘Constance South’, COS, and ‘Constance West’, COW; [44]; see also [68]). The majority of the study sites, however, have not been investigated before. The new systems include ‘Constance North’ (CON) and ‘Constance East’ (COE). In the latter, the stream site was sampled at two different locations (Grasbeuren, 7.6 km from the lake, and Mühlhofen, 4 km from the lake). These samples proved very similar phenotypically and genetically (e.g., F_ST_ = 0.002, P = 0.40; further details not presented), so that they were pooled to represent a single stream site (COE stream). Further, we sampled an additional stream for the COS system (‘COS1 stream’). Because this stream drains into LC at almost the same location as COS2 stream, these two systems share their lake counterpart.

**Table 1:** Localities, geographical coordinates, sampling year, and sample size for the five lake-stream stickleback systems in the Lake Constance basin (CON, COE, COS1, COS2, COW), and the two solitary stream populations (RHI, DAN).

Locality	System or site code	Habitat	Latitude (North)	Longitude (East)	Sampling year	Sample size
Iznang (DE)	CON	lake	47°43′3.36″	8°57′42.48″	2011	22 (10/12)
Bohlingen (DE)	CON	stream	47°43′18.84″	8°53′01.68″	2011	23 (15/7)
Unteruhldingen (DE)	COE	lake	47°43′25.32″	9°13′37.56″	2011	33 (18/15)
Grasbeuren (DE)	COE	stream	47°43′39.72″	9°18′23.4″	2011	13 (9/4)
Mühlhofen (DE)	COE	stream	47°44′11.76″	9°15′49.68″	2011	12 (7/5)
Fussach (AT)	COS1 & COS2	lake	47°29′29.7″	9°39′40.37″	2008	24 (3/21)
Hohenems (AT)	COS1	stream	47°21′18.55″	9°40′10.22″	2008	25 (11/14)
Rankweil (AT)	COS2	stream	47°16′19.28″	9°35′32.72″	2008	24 (12/12)
Romanshorn (CH)	COW	lake	47°33′22.5″	9°22′48.25″	2008/2009	24 (12/12)
Niederaach (CH)	COW	stream	47°33′29.25″	9°16′42.38″	2008/2009	25 (11/14)
Basel (CH)	RHI	stream	47°32′44.34″	7°33′51.84″	2011	24 (12/12)
Kirchbierlingen (DE)	DAN	stream	48°14′04.03″	9°43′30.86″	2011	34 (15/19)

The localities are situated in Germany (DE), Austria (AT), and Switzerland (CH). Sample sizes are total, and males and females in parentheses. Note that the same lake sample was used for both the COS1 and COS2 system, and that the COE stream site combines two samples (for details see text).

The origin of stickleback in the LC basin is unknown, but commonly attributed to human introduction (e.g., [44], [69]). The first report of the species’ wide-spread occurrence within the basin dates back to the mid 19^th^ century ([70], p. 320). To obtain new genetic insights into the populations’ possible origin, we complemented our paired lake-stream samples by samples from two solitary (allopatric) stream-resident populations. The first solitary population was sampled from a small creek draining into the River Rhine (the outlet stream of LC, draining into the Atlantic) near Basel, Switzerland (Fig. 1, Table 1). This sample is hereafter called the Rhine (RHI) sample. A recent study indicates strong differentiation in neutral markers between stickleback occurring in the Rhine catchment downstream of LC and the lake itself [69], suggesting that the latter was not colonized via the Rhine. Our Rhine sample allowed an independent evaluation of this hypothesis. The second solitary stream population (DAN) was sampled in the headwaters of the Danube River drainage near Kirchbierlingen, Germany. This sample was included because of the close proximity of the Danube drainage to the LC basin, and because the LC region drained into the Danube (and eventually into the present-day black sea region) in postglacial times [71].

All new samples were collected in the spring 2011 (late April, May; i.e., during the stickleback breeding season). The samples taken in previous years, and a few specimens collected in 2012 exclusively for the analysis of fecundity and egg size (see below), were also collected within that seasonal time frame. All samples were taken with permission from the corresponding fisheries authorities (Austria: Landesfischereizentrum Vorarlberg, A. Lunardon; Germany: Fischereiforschungsstelle Baden-Württemberg, S. Blank, M. Bopp, C. Wenzel; Switzerland: Jagd- und Fischereiverwaltung Thurgau, R. Kistler; Amt für Umwelt und Energie Basel-Stadt, H.-P. Jermann). Sampling occurred on breeding grounds using unbaited minnow traps. All individuals used for this study were in reproductive stage because the males consistently displayed breeding coloration, and gravid females were frequent at every site. The specimens were euthanized with an overdose of MS-222, taking all efforts to minimize suffering, and immediately weighed, photographed with a reference scale as described in [12], and stored in absolute ethanol. For most sites, a minimum sample of 12 individuals per sex could be achieved (Table 1). Unless noted otherwise, all analyses are based on the full sample from a given site. All work in this study was approved by the Veterinary Office of the Canton of Basel-Stadt (permit number: 2383).

### Analysis of Lake-stream Divergence in Life History

Our prime interest was to investigate lake-stream divergence in age and size at reproduction. To quantify age at reproduction, we retrieved the left and right sagittal otolith from all specimens in each lake-stream pair. The otoliths were cleaned mechanically using fine forceps, dried, mounted in 20 µl Euparal on a microscope slide, and inspected under a stereomicroscope at 50x magnification by a single person (DM) blind to the specimens’ origin. Illumination was from above on a black background to optimally visualize the opaque and transparent ring zones used for age determination following [72] (representative otoliths from different age classes are shown in Appendix S1). Left and right otoliths always produced consistent results. A total of 4 specimens (<2% of all specimens investigated) displayed unclear otolith ring patterns and could thus not be aged unambiguously. Excluding these specimens from analysis did not affect any conclusions; hence we present results based on the full data set. Differences in age composition between lake and stream fish were tested separately for each system through non-parametric permutation tests randomizing the response variable (age) 9999 times over the predictor (habitat) [73], and using the lake-stream difference in average age as test statistic. All statistical inference in this study is based on analogous permutation tests.

To quantify body size at reproduction, we digitized 16 homologous landmarks [44] on the photograph of each specimen by using TpsDig [74]. TpsRelw [74] was then used to calculate centroid size from the landmark configurations. This size metric, hereafter referred to as ‘body size’, was considered more robust to variation in overall body shape and feeding or reproductive status than size metrics such as standard length or linearized body mass. (Using the latter as body size metric, however, produced very similar results in all analyses.) To test for lake-stream divergence in body size, we used the difference in average size between the habitats as test statistic.

In addition to age and size at reproduction, we investigated divergence in fecundity and egg size. For this, clutches of gravid females ready for spawning were collected in the field by gently squeezing the females’ abdomen, and preserved in ethanol. We then counted the total number of eggs (fecundity) under a stereomicroscope, dried all eggs at 50°C for 48 h, and determined their total dry mass. Egg size was then expressed as the total clutch dry mass divided by total egg number (i.e., the average dry mass of a single egg). This investigation used mainly females collected in 2012 for this specific purpose only (and hence not included in Table 1; lake: COE, COW, N = 11 each; stream: COW, CON, COE, N = 9, 1, 1), but additionally involved a few females also used for the other analyses (details given in Table S1). Testing for lake-stream divergence in fecundity and egg size was then performed in a single analysis for each trait by pooling data across the two lake sites and the three stream sites. (Restricting the analysis to the COW system with sufficient data from each habitat produced similar results.) As above, the difference in trait means between the habitats was used as test statistic.

### Comparison of Body Size Among Global Populations

To interpret the body size patterns revealed in our lake-stream and solitary stickleback populations from Central Europe in a broader geographic and ecological context, we performed a comparison of reproductive body size by including a total of 21 additional stickleback populations from different geographic regions and habitats. We hereafter call this the ‘global’ data set, acknowledging that these samples do not represent the species’ full body size diversity (e.g., [52]). These additional samples comprised lake populations from Beaver, Boot, Joe’s, Misty, Morton, Pye, and Robert’s Lake (sites described in [43]), and from Hope Lake (coordinates: 50°34′0″ N, 127°20′30″ W), on Vancouver Island (British Columbia, Canada). Additional stream-resident populations were from the Beaver, Boot, Joe’s, McCreight, Pye, and Robert’s systems [43], and from the inlet stream to Misty Lake [39], [75], on Vancouver Island. These freshwater samples were complemented by collections of marine stickleback from two estuaries on the east coast of Vancouver Island (Cluxewe: 50°36′51″ N, 127°11′10″ W; Sayward [76]), from the Japan Sea and Pacific [77], from the Atlantic Coast in Norway [78], and from the coast of the White Sea in Russia [79]. All these additional samples were also collected during the reproductive season on breeding grounds. Body size was quantified from available photographs as described above. Sample size was 20–36 individuals per site, with both sexes well represented.

For the global comparison of body size at reproduction, we first pooled all samples from the LC basin within each habitat type. This was done to avoid pseudo-replication, and because body size within each habitat type was highly consistent (see below). Interestingly, visual inspection of the data from the global samples suggested differences among the three habitat types (lake, stream, marine) in the *variability* of average body size across populations. This was tested formally through separate lake-stream and marine-stream tests using the variance in population means as test statistic.

### Additional Phenotypic Analyses

The above analyses were complemented by investigating two additional variables potentially relevant to life history evolution. First, as life history divergence might be driven by differential food resources, we analyzed prey items in stomachs of stickleback from one system (COW lake and stream; N = 20 and 7). Because lake stickleback might exploit different prey resources during the reproductive period spent in littoral (near-shore) breeding habitat than during non-reproductive life stages (e.g., [80]), we additionally acquired a small sample (N = 5) of stickleback caught by LC fishermen in offshore drift nets targeting pelagic whitefish. This sample was taken off the COS lake site in April 2011. To ensure adequate quality of stomach content for analysis, all specimens (lake offshore, lake littoral, and stream) were preserved within 5 h upon setting the capturing device (minnow trap, drift net). Prey items were identified to order, family, or genus, and assigned to broad taxonomic groups (e.g., pelagic cladocera, vermiform insect larvae; see Table 2). For every stickleback, we determined the relative proportion of the total prey items accounted for by each taxonomic group, calculated summary statistics for each of the three habitat types, and interpreted these statistics qualitatively. This approach was preferred to a formal analysis because of the relatively small sample sizes.

**Table 2:** Stomach content of stickleback from the Lake Constance offshore site, and from the lake and stream site in the COW system.

	Pelagic	Pelagic or benthic	Benthic				
	Cladocera^1^	Copepods	Cladocera^2^	Other crustacea^3^	Vermiform insect larvae^4^	Other insect larvae^5^	Stickleback eggs
Lake offshore	0.34 (0.21)	0.66 (0.21)	–	–	–	–	–
COW lake	0.01 (0.02)	0.07 (0.1)	0.33 (0.29)	0.03 (0.08)	0.42 (0.37)	0.15 (0.24)	0.03 (0.11)
COW stream	–	0.17 (0.18)	0.2 (0.25)	–	0.57 (0.27)	0.06 (0.08)	0.09 (0.2)

1 Daphnia, Ceriodaphnia, Bosmina.

2 Chydoridae.

3 mainly Ostracoda.

4 Chironomidae, Ceratopogonidae.

5 mainly Ephemeroptera and Plecoptera.

The values represent the proportion of the total prey items accounted for by each prey class, averaged across individuals within each site (standard deviation in parentheses). The copepods category subsumes pelagic, benthic, and/or generalist taxa difficult to distinguish; strictly pelagic calanoid copepods, however, were found in the offshore lake specimens only. Sample size is 5, 20, and 7 for offshore, COW lake, and COW stream.

The second additional variable was the lateral plate phenotype. Ancestral marine stickleback are protected from vertebrate predators in their pelagic environment by bony lateral plates along their entire body [81]. This phenotype is disfavoured in most freshwater environments, as stickleback in lakes and streams generally display an adaptive, genetically-based reduction in the number of lateral plates [81]. We considered this trait here because the major genetic factor determining plate phenotype (the ectodysplasin gene, *EDA*; [82]) might pleiotropically influence growth rate [83], and because stickleback in the LC basin are polymorphic for both plate phenotype and the underlying *EDA* alleles [44]. Following this latter study, we assigned all individuals to one of three lateral plate phenotype morphs (full, partial, low). We then tested for lake-stream divergence in plate morph frequency within each system by using the Chi-square ratio as test statistic (extending similar tests already performed for the COW and one of the COS systems; [44]). Next, sufficiently polymorphic samples (i.e., the stream samples of CON, COE, and COW) were used to test for an association between plate morph and body size by using the F ratio from analysis of variance as test statistic [73]. All statistical analyses and plotting were performed in R ([84]; codes available on request). All phenotypic data are provided in Table S1.

### Genetics

The major goal of our genetic investigation based on nuclear and mitochondrial markers was to quantify population structure within and among the replicate lake-stream systems in the LC basin. Of particular interest was the detection of strong genetic divergence within lake-stream systems, suggesting effective habitat-related barriers to gene flow. An additional goal was to explore the relationship between stickleback in the LC basin and fish from nearby water bodies. The present work greatly extends a previous population genetic study partly involving fish from the LC basin [44] in that new lake-stream pairs are analyzed, samples from the Rhine and Danube are included, and a greater number of genetic markers are used.

We first extracted DNA from pectoral and caudal fin tissue on a MagNA Pure LC extraction robot (Roche) by using the Isolation Kit II (tissue). Next, we amplified eight microsatellites with labelled primers in two separate multiplex PCRs by using the QIAGEN multiplex kit and following the manufacturer’s protocol. All PCRs included a negative control to check for contamination. The microsatellite markers were chosen to be far from known quantitative trait loci in stickleback, and to lie on different chromosomes. They included the markers Stn67, Stn159, Stn171, and Stn195 used previously [12], [44], and additionally Stn28, Stn99, Stn119, and Stn200 [85]. For the latter, we designed our own primer pairs (primer sequences for all eight markers are provided in Table S2). PCR products were run on an ABI3130*xl* sequencer (Applied Biosystems), and alleles scored manually in PeakScanner v1.0. Input files for the different population genetic programs were prepared by using CREATE [86].

The microsatellite data were first used to estimate differentiation among all 11 samples by Weir & Cockerham’s F_ST_ [87] calculated with GENETIX v4.0.5.2 [88] (P-values based on 999 permutations). To account for variation in heterozygosity within populations [89], we also calculated *standardized* F_ST_ after data transformation with RECODEDATA v0.1 [90]. Next, we tested whether neighboring lake and stream samples qualified as genetically distinct populations by performing a genetic clustering analysis using STRUCTURE (v2.3.1; [91], [92]) separately in each lake-stream pair (note that the COS system represents two pairs, both involving the same lake sample). The assumed number of populations (K) ranged from one to three, with each level replicated five times under the admixture and independent allele model with 100’000 iterations (20′000 iterations burnin). An additional analysis examined population structure among the 11 pooled samples, using K = 1–12. STRUCTURE results were combined using Structure Harvester v.0.6.92 [93], and interpreted following [94], [95]. The microsatellite data set is provided in Table S3.

The above analyses using rapidly evolving microsatellites were complemented by a more coarse-grained investigation of genetic relationships based on single nucleotide polymorphisms (SNPs) within a 305 bp segment of the mitochondrial D-loop. Sample size was 18–32 individuals per site, 256 in total. Primers and PCR amplification conditions were as in [44]. Products were sequenced on an ABI3130*xl* sequencer (Applied Biosystems). We used jModelTest v0.1.1 [96] to determine the most appropriate model of sequence evolution (‘F81’; [97]), identified the most probable genealogical relationship by the maximum-likelihood method implemented in PAUP* v4.0 [98], and generated a haplotype genealogy for visualization following [99]. All D-loop sequences are deposited in GenBank (accession numbers JX436521-JX436776).

## Results

### Phenotypic Analyses

**Figure 2:**
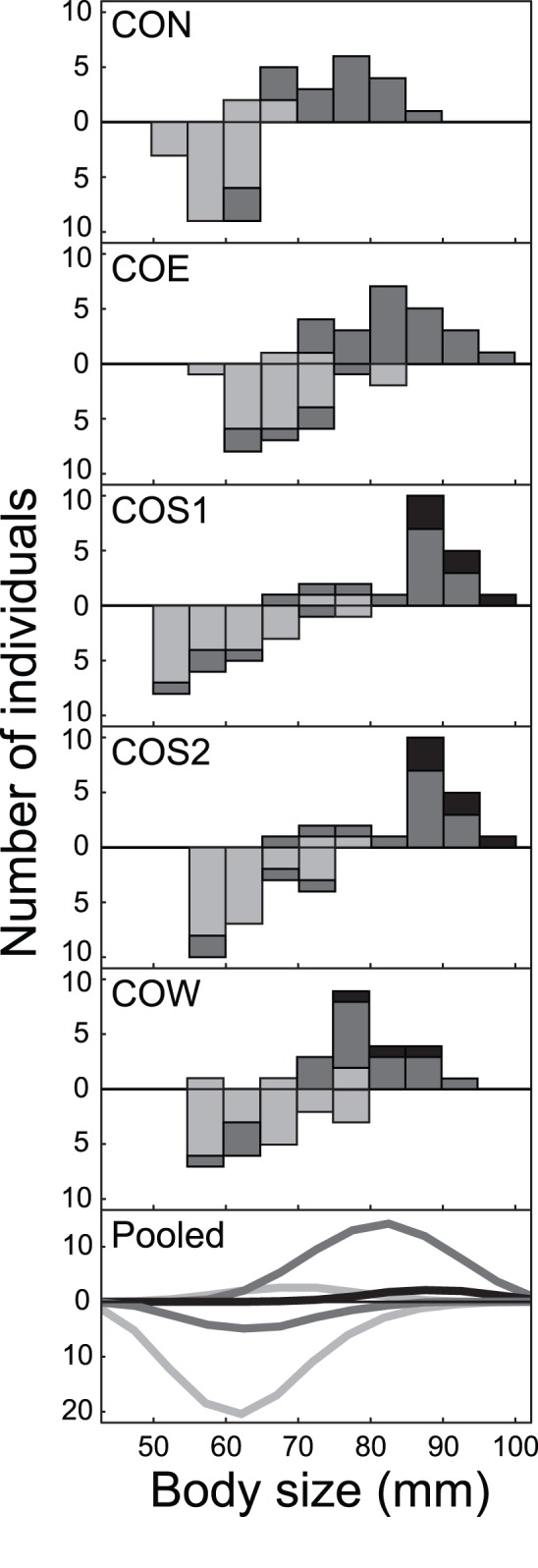
Age and body size at reproduction in lake and stream stickleback from the Lake Constance basin. The top panels show body size (quantified as landmark-based centroid size) histograms for each lake-stream system separately, with the lake data pointing upward and the stream data pointing downward. Proportions are shaded according to age class; individuals in their second, third, and fourth calendar year are drawn in light gray, dark gray, and black. The bottom panel follows the same drawing conventions, except that here the data are pooled across all systems within each habitat type, and smoothed by LOESS (locally weighted scatterplot smoothing) for each age class separately. Note the striking shift toward greater age and size at reproduction in lake stickleback as compared to their conspecifics from streams.

The otolith analysis revealed strong and highly consistent lake-stream divergence in age at reproduction in all replicate systems in the LC basin (all P<0.0015). Generally, stickleback on breeding grounds in the lake were in their third calendar year (i.e., approximately two years old), with a few individuals breeding in their second or fourth calendar year (Fig. 2). By contrast, stream stickleback essentially displayed an annual life cycle; individuals in their third calendar year were rare, and no single fish was found to breed in its fourth calendar year.

**Figure 3:**
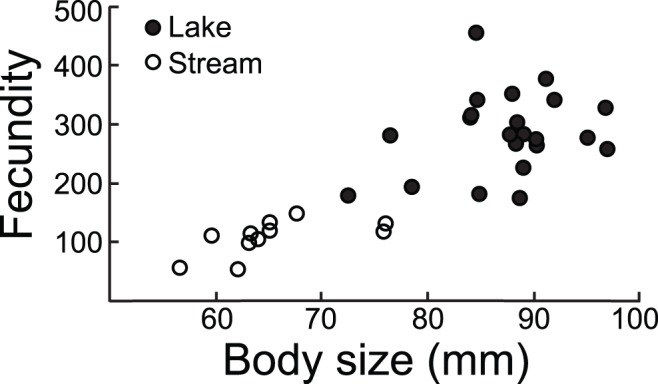
Fecundity in relation to body size in female stickleback from Lake Constance and its tributary streams. Fecundity is expressed as number of eggs per clutch. Within each habitat class, samples were pooled across different locations (lake: N = 22; stream: N = 11).

Lake-stream shifts in age at reproduction were paralleled by strong divergence in body size, with lake fish on average exhibiting 27% greater size than stream fish (lake mean centroid size across all systems: 80.4 mm; stream: 63.2 mm; P = 0.0001 in all systems) (Fig. 2). Translated to fresh body mass, the average size difference was more than twofold (lake: 2.53 g; stream 1.19 g; a photograph of a representative lake and stream individual is shown in Appendix S1). Body size divergence was further associated with dramatic divergence in fecundity (Fig. 3): on average, the (larger) lake females displayed a threefold higher number of eggs than the stream females (284 *versus* 94; P = 0.0001). Egg size, however, did not differ between the habitats (P = 0.51).

**Figure 4:**
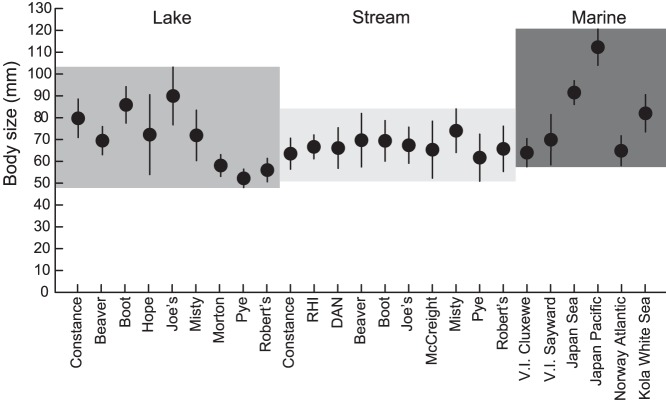
Body size at reproduction in the global stickleback populations from lake, stream, and marine habitats. Samples from the Lake Constance basin are pooled for each habitat type (further details on the samples are given in the text). Error bars are one standard deviation in each direction. The shaded boxes behind the symbols indicate the body size range spanned by the standard deviations in each habitat. Note the low variance in population mean size among the stream populations as compared to lake and marine fish.

Our comparison of body size across global stickleback samples from lakes, streams, and the sea indicated a clear difference in the variance in population average size among the habitats. Strikingly, all stream populations investigated displayed relatively similar average size, whereas the lake samples were much more variable (lake-stream difference in variance: P = 0.002; Fig. 4). The latter included very small-bodied populations (Morton, Pye, and Robert’s) as well as large-bodied populations (Boot, Joe’s). Body size among marine stickleback also tended to be more variable than among stream populations (marine-stream difference in variance: P = 0.065; note the small sample size for marine fish, and hence low statistical power in this test).

In addition to the above life history patterns, our analysis of stomach content revealed a very clear difference in prey utilization by lake and stream stickleback, despite the modest sample sizes. In particular, our pelagic sample showed clearly that LC stickleback forage on zooplankton outside the breeding grounds; the stomachs of these specimens contained exclusively small pelagic crustacea (Table 2). By contrast, the stomachs of the stream fish contained exclusively benthic prey (predominantly chironomid larvae and benthic cladocera), highly consistent with data from streams on Vancouver Island [43]. Similar benthic prey was also found in the lake fish collected on (littoral) breeding grounds, indicating a reproductive shift in foraging mode in stickleback residing within LC.

In all three new lake-stream systems subjected to lateral plate morph analysis (CON, COE, COS1), we found a trend toward plate reduction in the stream as compared to the lake where fully plated fish predominated clearly. The shift in plate morph frequency was particularly striking in the COE system (P = 0.0001), paralleling a similar pattern found previously in the COW system [44] (details given in Appendix S1). However, we found no relationship between plate morph and body size at reproduction in any of the three investigated stream samples (CON, COE, COW; all P> = 0.35).

### Genetics

A striking pattern revealed by our eight microsatellite markers was the absence of population structure among the four geographically distant LC samples. None of the six total pairwise F_ST_ values among these lake samples exceeded 0.01 (all P> = 0.07) (Table 3). Genetic differentiation *within* the lake-stream pairs was mostly modest as well, but sometimes reached substantial values despite a much shorter geographic distance between the paired lake and stream sites than among the lake sites (COE: F_ST_ = 0.18, P = 0.001; COS2: F_ST_ = 0.08, P = 0.001). Microsatellite differentiation among the stream samples was generally substantial, with F_ST_ averaging 0.10 (all P<0.004 except CON-COS1, P = 0.13). Furthermore, our Rhine sample (RHI) displayed strong differentiation from all samples in the LC basin (F_ST_ = 0.16–0.29), whereas differentiation between the Danube sample (DAN) and stickleback from the LC basin was rather low. For instance, all five comparisons between DAN and LC samples produced F_ST_ < = 0.04 (P = 0.001–0.023).

**Table 3:** Pairwise genetic differentiation among the nine lake and stream stickleback samples from the Lake Constance basin, and the two solitary samples, based on eight microsatellite markers.

	CONlake	CONstream	COElake	COEstream	COSlake	COS1stream	COS2stream	COWlake	COWstream	RHI	DAN
CON lake		0.00(0.676)	0.01 (0.071)	0.18 **(0.001)**	0.01 (0.240)	0.02 (0.041)	0.10 **(0.001)**	0.00(0.305)	0.05 **(0.001)**	0.27 **(0.001)**	0.03 **(0.002)**
CON stream	0.00		0.00 (0.587)	0.15 **(0.001)**	0.00 (0.386)	0.01 (0.132)	0.06 **(0.001)**	0.00 (0.759)	0.03 **(0.004)**	0.25 **(0.001)**	0.02 (0.011)
COE lake	0.02	0.00		0.18 **(0.001)**	0.00 (0.543)	0.02 **(0.003)**	0.07 **(0.001)**	0.00 (0.744)	0.04 **(0.001)**	0.28 **(0.001)**	0.03 **(0.001)**
COE stream	0.55	0.46	0.50		0.20 **(0.001)**	0.17 **(0.001)**	0.21 **(0.001)**	0.17 **(0.001)**	0.13 **(0.001)**	0.16 **(0.001)**	0.17 **(0.001)**
COS lake	0.02	0.00	0.00	0.56		0.01 (0.160)	0.08 **(0.001)**	0.00 (0.478)	0.03 **(0.001)**	0.28 **(0.001)**	0.04 **(0.001)**
COS1 stream	0.05	0.02	0.05	0.47	0.02		0.06 **(0.001)**	0.02 (0.053)	0.03 **(0.002)**	0.24 **(0.001)**	0.08 **(0.001)**
COS2 stream	0.22	0.13	0.15	0.52	0.17	0.12		0.08 **(0.001)**	0.11 **(0.001)**	0.29 **(0.001)**	0.12 **(0.001)**
COW lake	0.00	0.00	0.00	0.48	0.00	0.05	0.17		0.02 **(0.007)**	0.26 **(0.001)**	0.02 (0.023)
COW stream	0.13	0.08	0.10	0.40	0.07	0.07	0.25	0.05		0.21 **(0.001)**	0.06 **(0.001)**
RHI	0.69	0.64	0.66	0.46	0.66	0.56	0.62	0.62	0.54		0.26 **(0.001)**
DAN	0.08	0.05	0.07	0.50	0.00	0.19	0.27	0.05	0.16	0.65	

The upper semimatrix gives Weir & Cockerham’s F_ST_ estimator [87], with P-values based on 999 permutations in parentheses (bold if P<0.01). The lower semimatrix presents F_ST_ standardized by the maximum differentiation possible given the observed magnitudes of within-population heterozygosity [89].

The results from the STRUCTURE analysis agreed well with the F_ST_-based patterns. First, analyzing each system separately, STRUCTURE identified the system displaying the highest lake-stream differentiation (COE) as consisting of two genetically distinct populations. The four other systems qualified as a single population (details not presented). Analyzing all 11 samples together suggested two distinct genetic clusters. The first cluster involved RHI and the stream site of COE, the second involved all other populations from the LC basin plus the DAN sample. However, the STRUCTURE algorithm can perform poorly when faced with highly imbalanced sample sizes [100]. Indeed, most samples from the LC basin were genetically so similar that they essentially formed one single large sample, which probably caused RHI and COE stream to cluster together despite strong genetic differentiation (F_ST_ = 0.16). However, when analyzing only RHI, COE stream, and a *single* lake sample together, three distinct populations were indicated, as expected based on F_ST_.

**Figure 5:**
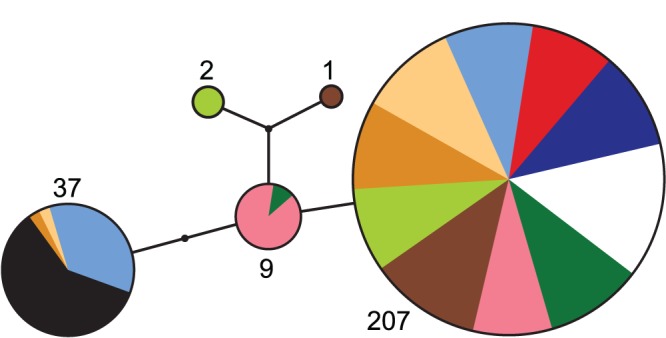
Haplotype network for the lake-stream stickleback pairs in the Lake Constance basin and the solitary populations. The network is based on six single nucleotide polymorphisms in the mitochondrial D-loop. The numbers give the total count for each haplotype. Color codes are as in Fig. 1.

Our mitochondrial D-loop sequencing identified six total SNPs, defining five distinct haplotypes (Fig. 5). One of these haplotypes was clearly predominant; it was either the only one discovered, or at least very frequent, in *all* samples from the LC basin. Notably, this haplotype was also the only one found in the DAN sample. By contrast, all individuals from RHI exhibited a different haplotype shared only with some individuals from three stream samples of the LC basin. Three additional haplotypes occurred at low frequency, mainly in stream fish.

## Discussion

### Life History Divergence and Implications for Reproductive Isolation

Divergence in life history traits might strongly contribute to reproductive isolation, and yet its role in speciation is little explored. We here investigated life history in stickleback residing in Lake Constance and multiple tributary streams, revealing dramatic divergence between the two habitats: lake fish reproduce at much greater age and size than their conspecifics in the streams, and these patterns coincide with much greater fecundity in females from the lake. These findings parallel concurrent shifts in age and size at reproduction and in reproductive investment reported from North American lake populations [52], [55], [56]. The only life history trait that proved stable between lake and stream stickleback was egg size, possibly indicating similar stabilizing offspring viability selection in both habitats [101], [102].

Divergence in age and size at reproduction was highly consistent across multiple replicate habitat pairs in the LC basin, and our genetic data indicate clearly that this results from repeated evolution in stream stickleback. The reason is that the stream samples consistently displayed strong mutual microsatellite differentiation, contrary to the lake samples exhibiting negligible differentiation. This pattern clearly rules out the possibility that the different stream populations originate from a common ancestral stream stickleback population. Moreover, the rare D-loop haplotypes found in the LC basin were mostly unique to specific stream samples (Fig. 5), consistent with independent founder events (i.e., haplotype frequency shifts caused by strong genetic drift in the small stream founder populations). Together, our life history and genetic data thus argue strongly for the independent colonization of the different tributaries by an essentially panmictic LC population, followed by repeated life history evolution in stream stickleback.

Given the great magnitude of lake-stream divergence in body size, and the general importance of this trait in mate choice and male aggressive interactions in the species [59]–[61], [63]–[66], the observed life history shifts might well contribute to reducing gene flow across the lake-stream habitat transitions. Indeed, our F_ST_-based analysis revealed substantial lake-stream differentiation within some systems (with values reaching 0.18), and STRUCTURE identified two distinct populations in one of them. This allows us to infer the presence of strong reproductive barriers at a small spatial scale, consistent with findings from lake-stream systems in Pacific North America [12], [46], [49], [50]. Note that the *weak* marker divergence seen in some of our systems (CON, COS1; F_ST_ < = 0.01) does not conflict with this conclusion; because the colonization of the LC basin is presumably relatively recent (see below), detecting reproductive isolation with neutral markers is expected to be difficult [44], [103]. The presence of effective habitat-related reproductive barriers is also supported by the consistent and sometimes substantial (COE, COW) lake-stream divergence in plate morph frequency (Appendix S1). This divergence has a strong genetic basis [44] and would not have arisen, or be maintained, in the absence of effective barriers to gene flow. Nevertheless, the extent to which the observed lake-stream shifts in life history actually contribute to reproductive isolation cannot be evaluated based on the present data.

### Mechanisms of Life History Divergence

In many organisms, the transition of resource allocation from growth to reproductive life is governed by critical maturation size thresholds (reviewed in [104], [105]). Although not investigated in detail, this seems to hold for stickleback as well [106], [107]: as long as an individual has not attained this threshold, environmental cues signalling spring conditions will not trigger maturation and reproductive behavior. On the basis of this maturation control, we propose two not mutually exclusive hypotheses explaining life history divergence in lake-stream stickleback in the LC basin. First, assuming similar growth rates in both habitats, lake fish might exhibit a relatively higher maturation size threshold (due to genetic divergence and/or phenotypic plasticity) that they generally cannot attain within one year. Only after two years of growth, lake fish would exceed their maturation threshold and start reproducing – and at that time also be much larger than the stream fish reaching their threshold size within one year [105]. This hypothesis is plausible: body size divergence among populations of *ninespine* stickleback is attributable to genetically-based divergence in maturation size thresholds [108], [109].

Alternatively, maturation size thresholds might be similar among the populations, but growth rates might be lower in lake fish than in tributary stream populations (again due to genetic divergence, phenotypic plasticity, or both). The consequence would be the same as above: lake fish would require two years of growth to attain their maturation threshold, but mature larger [105]. Indeed, our study provides evidence of differential growth rates between the habitats. As the analysis of stomach content suggests, stickleback inhabiting LC exploit exclusively zooplankton prey outside the breeding grounds. These fish are also an occasional by-catch in off-shore drift nets (personal communications from LC fishermen), and are absent from littoral habitat outside the breeding season (D. Moser, personal observation). Moreover, for a freshwater population, stickleback in LC display extremely long gill rakers [44], a character state generally associated with zooplankton exploitation [110] and typical of pelagic marine stickleback [76]. Stickleback residing within LC thus display a pelagic life style, with a foraging niche shift during the reproductive period (see also [80]). Note also that the LC fish provide a rare example of a freshwater population almost fixed for the full lateral plate morph (Appendix S1), a phenotype presumably favored in pelagic populations highly exposed to vertebrate predation [111]. (We found no evidence, however, for a direct relationship between plate phenotype and life history traits.).

By contrast, stream populations in the LC basin exploit exclusively benthic resources. Within the LC basin, we thus find similarly strong divergence in foraging modes as seen in the most ecologically divergent lake-stream pairs on Vancouver Island, Canada [12], [43], [49]. This difference in resource use might directly induce differential growth performance between the habitats, as benthic foraging generally seems to allow for a higher growth rate than pelagic foraging [112], [113]. Direct evidence for divergence in growth rates comes from a small sample of juvenile stickleback captured during the breeding season at the edge of the breeding ground at the COE lake site (non-reproductive status was confirmed by dissection; testes and ovaries were poorly developed). These fish displayed body sizes clearly below those of stream stickleback (43–49 mm, N = 3), and yet otolith analysis confirmed that they were already one year old (data presented in Table S1). It thus appears plausible that a lower growth rate in lake stickleback, induced by a relatively poor pelagic resource base, underlies the lake-stream divergence in life history observed within the LC basin (acknowledging the possibility that differential growth rates in the two habitats has a genetic component).

The direct induction or genetically based evolution of an annual life cycle in response to more profitable benthic resources in streams would explain the relatively low variance in average body size across stream populations from different geographic regions (Fig. 4). The reason is that the resource spectrum used by stream stickleback is highly consistent across global populations, while lake populations are more variable in resource use [12], [43], [49], [114]. If variation in population mean size was (at least partly) a consequence of resource-dependent variation in growth rate, we would indeed expect lake population means to be more variable than stream means. We note, however, that *small-sized* lake populations are not necessarily benthic-foraging. For instance, the lake population with the smallest average size in Fig. 4 (Pye Lake, Vancouver Island) exploits a strictly pelagic food base [43]. Hence, factors other than food resources (e.g., predation [57], [58]) likely contribute to the presumably greater life history diversity in lake (and perhaps marine) stickleback than in stream stickleback.

Body size divergence through resource-mediated plasticity in growth rate might play a particularly important role in reproductive isolation. The reason is that this divergence would occur, and potentially influence sexual interactions, within a *single* generation after the colonization of a stream by lake fish [35], [36]. It would therefore be crucial to quantify environmental and genetic contributions to life history divergence in stickleback from the LC basin and elsewhere.

### Origin of Stickleback in the Lake Constance Basin

Consistent with a previous population genetic investigation [69], our genetic analyses indicate that the populations in the LC basin do not originate from colonization by stickleback residing in the Rhine downstream of LC. However, we find that stickleback in the LC basin are genetically very closely related to those occurring in the nearby Danube drainage: pairwise differentiation between Lake Constance samples and DAN was consistently low (F_ST_ < = 0.04), and the only D-loop haplotype found in DAN was the one also predominant in the LC basin. Is it possible that LC stickleback derive from a source population from the Black Sea region that colonized naturally via the Danube? A population genetic study in European perch (*Perca fluviatilis*) [71] and geological data [115] suggest the existence of such a temporary colonization route during the last glacial retreat. In fact, a connection between the Danube drainage and the LC basin still persists today, as the source of the stream sampled at the CON stream site is formed by water captured from the Danube headwaters through a sinkhole and a 12 km underground stream [116]. Whether this allows for fish dispersal has not been investigated.

A scenario of colonization via the Danube, however, is challenged by the absence of stickleback from the entire Danube drainage reported in the nineteenth century ([70], p. 319; the species was already present in the LC basin at that time), although the reliability of this information is unknown. Moreover, stream-resident stickleback are generally low-plated (e.g., [38], [42], [117]–[119]). The incomplete shifts toward the low-plated morph in our stream samples from the LC basin, along with the low haplotype diversity within the basin, might thus be taken as tentative support of a relatively recent origin, perhaps due to human introduction. More extensive phylogeographic data from Central and Eastern European populations are needed for a better understanding of the origin and age of stickleback in the LC basin and the Danube drainage.

### Conclusions

We have shown strong, repeated, and possibly rapid life history divergence between lake and stream stickleback in the Lake Constance basin, sometimes coinciding with substantial differentiation in neutral markers. Our comparison of body size patterns across global populations and habitats, combined with data from other stickleback systems, further suggests that life history divergence is very common in this species. Our study opens up several important avenues for further investigation: first, experimental work should uncover the mechanistic basis of life history shifts; are they due to differences in maturation size thresholds, in growth rate, or both? Second, the relative contribution of phenotypic plasticity *versus* genetic change to life history divergence should be quantified, and the ecological basis of divergence (e.g., contrasting trophic environments, differential predation regimes) should be identified. Finally, great efforts will be needed to understand whether life history divergence is primarily an aspect of adaptive divergence facilitated by already existing barriers to gene flow, or whether life history divergence itself is a major source of reproductive isolation between lake and stream populations.

## Supporting Information

Table S1 Sample site information and phenotypic data for all individuals.
(TXT)

Table S2 Primer sequences used to amplify the eight microsatellite markers.
(TXT)

Table S3 Microsatellite allele data for all individuals included in the population genetic analysis.
(TXT)

Appendix S1 Contains figures displaying representative stickleback otoliths of different ages, illustrating lake-stream divergence in body size, and summarizing lateral plate morph data for all study sites.
(PDF)
