# Supplementary material for: Repeated Lake-Stream Divergence in Stickleback Life History within a Central European Lake Basin
Source: PLoS One. 2012 Dec 4;7(12):e50620. doi: 10.1371/journal.pone.0050620 (PMC3514289; doi:10.1371/journal.pone.0050620)
Supplement: Appendix S1 — Contains figures displaying representative stickleback otoliths of different ages, illustrating lake-stream divergence in body size, and summarizing lateral plate morph data for all study sites. (PDF) [file pone.0050620.s004.pdf]

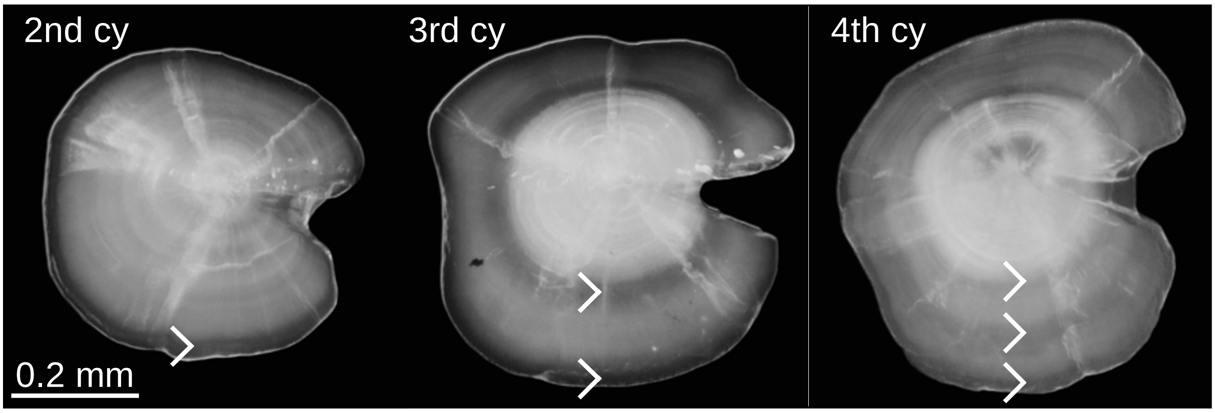


Representative sagittal otoliths of stickleback from Lake Constance in their second, third, and fourth calendar year (cy), photographed at 50x magnification. The dark (transparent) ring zones, accreted in spring [72] and used for age determination, are indicated by white arrows.

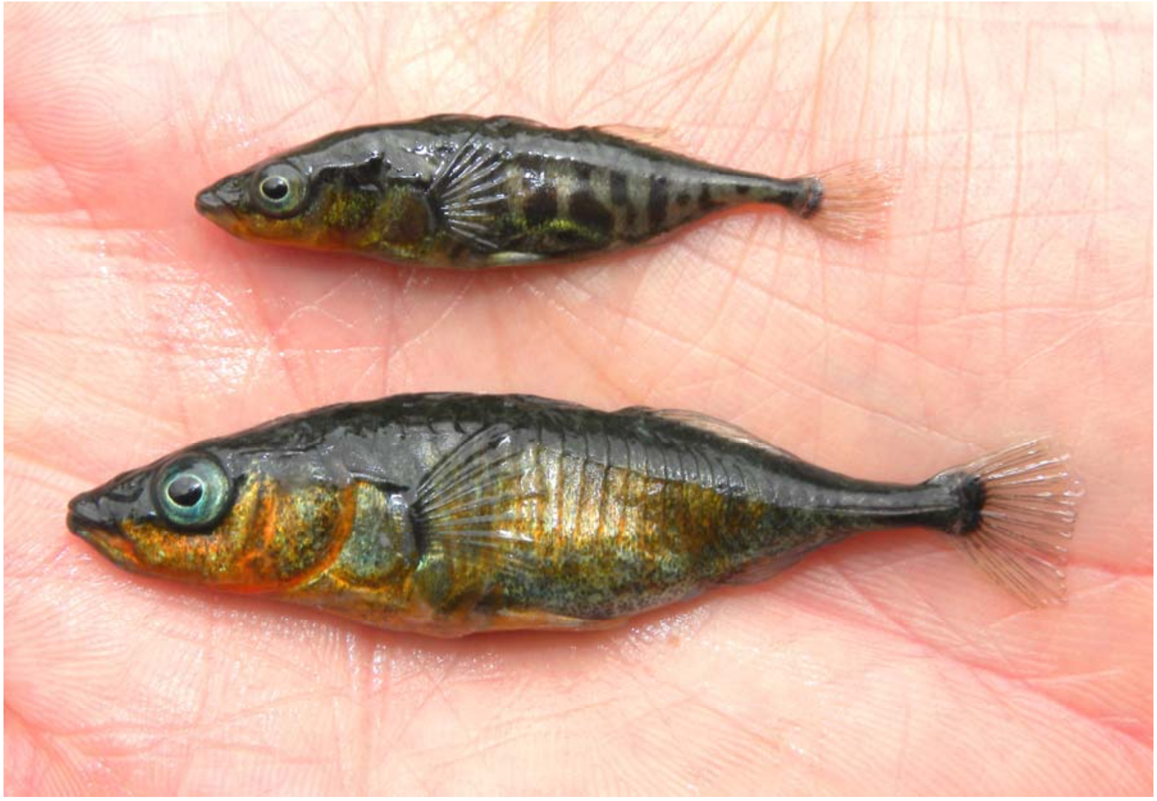

Representative male stickleback from COW lake (bottom) and COW stream (top). Photo credit: Daniel Berner.

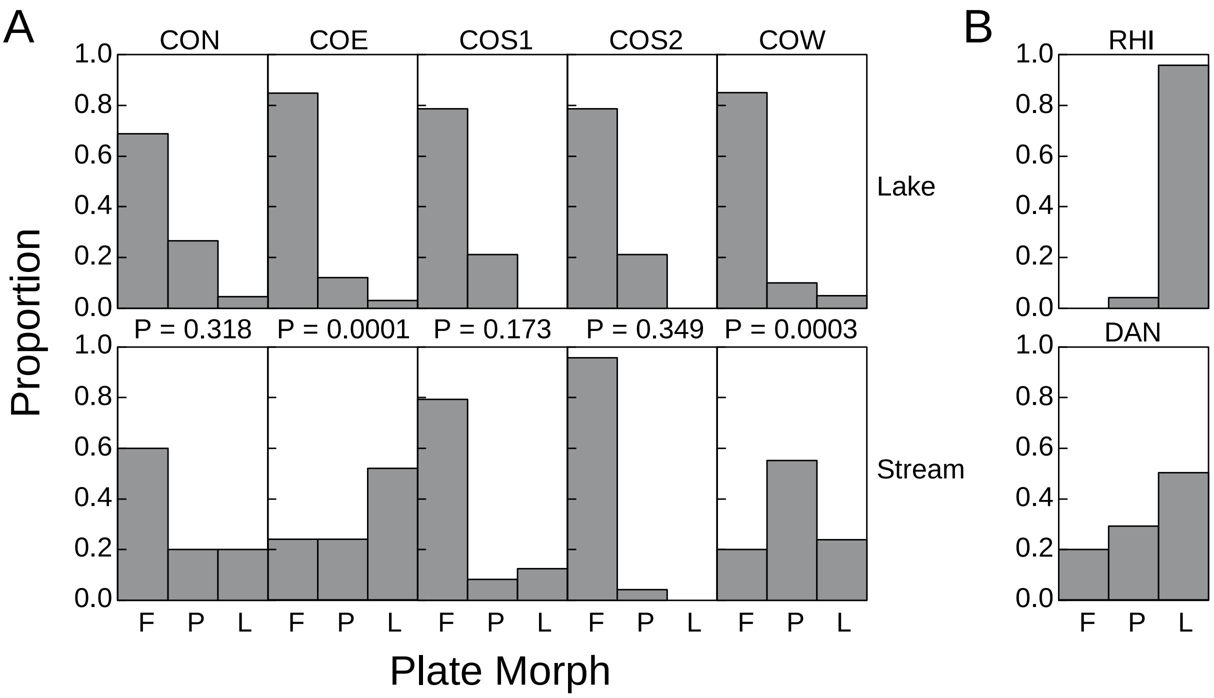


Proportion of fully (F), partially (P), and low-plated (L) stickleback morphs in the five lake-stream pairs (A; lake samples on top, stream samples on the bottom), and in the two solitary stream-resident populations (B). Sample site codes are given in Table 1. P-values are from permutation tests for lake-stream shifts in plate morph frequency within each system. Note the general trend toward plate reduction in the stream samples as compared to the lake samples. P-values and plate morph frequencies for the COW and COS2 system already investigated previously [44] are slightly different from those reported in that study because of random permutation, and because the present study analyzed subsamples of the previous study.
